# Romantic attachment, social support, sexual approach style and sociocultural influences on eating disorder symptoms

**DOI:** 10.1186/2050-2974-1-S1-O27

**Published:** 2013-11-14

**Authors:** Cassandra Dean, Janice Sabura Allen, Elizabeth Hughes

**Affiliations:** 1 School of Psychiatry and Psychology, Monash University, Australia; 2 Centre for Adolescent Health, The Royal Children's Hospital, Australia

## 

The purpose of this study was to examine the prediction of eating disorder symptoms within an integrated theoretically-driven model of romantic attachment, social support, sexual approach styles and the internalization of the media's portrayal of ideal body standards. A community sample of 671 women aged 19 to 88 years completed a series of self-report questionnaires. The findings supported a mediation model of romantic attachment being associated with eating disorder symptoms through interpersonal factors (perceived social support, game-playing and possessive sexual approach styles), and an environmental factor (the internalization of the media's depiction of ideal body standards). Romantic attachment anxiety predicted eating disorder symptoms directly; however, romantic attachment avoidance did not. Instead it predicted other factors. The findings enhance the understanding of the pathways influencing eating disorder symptoms and indicate that the attachment theory is a valuable framework to integrate the literature regarding sociocultural theory, sexual approach style, social support and eating disorders.

This abstract was presented in the **Disordered Eating – Characteristics & Treatment** stream of the 2013 ANZAED Conference.

